# Structural insight into substrate recognition by the endoplasmic reticulum folding-sensor enzyme: crystal structure of third thioredoxin-like domain of UDP-glucose:glycoprotein glucosyltransferase

**DOI:** 10.1038/srep07322

**Published:** 2014-12-04

**Authors:** Tong Zhu, Tadashi Satoh, Koichi Kato

**Affiliations:** 1 School of Physical Sciences, The Graduate University for Advanced Studies, 5-1 Higashiyama, Myodaiji, Okazaki, Aichi 444-8787, Japan; 2 Okazaki Institute for Integrative Bioscience and Institute for Molecular Science, National Institutes of Natural Sciences, 5-1 Higashiyama, Myodaiji, Okazaki, Aichi 444-8787, Japan; 3 Graduate School of Pharmaceutical Sciences, Nagoya City University, 3-1 Tanabe-dori, Mizuho-ku, Nagoya 467-8603, Japan; 4 JST, PRESTO, 3-1 Tanabe-dori, Mizuho-ku, Nagoya 467-8603, Japan

## Abstract

The endoplasmic reticulum (ER) possesses a protein quality control system that supports the efficient folding of newly synthesized glycoproteins. In this system, a series of *N*-linked glycan intermediates displayed on proteins serve as quality tags. The ER folding-sensor enzyme UDP-glucose:glycoprotein glucosyltransferase (UGGT) operates as the gatekeeper for ER quality control by specifically transferring monoglucose residues to incompletely folded glycoproteins, thereby allowing them to interact with lectin chaperone complexes to facilitate their folding. Despite its functional importance, no structural information is available for this key enzyme to date. To elucidate the folding-sensor mechanism in the ER, we performed a structural study of UGGT. Based on bioinformatics analyses, the folding-sensor region of UGGT was predicted to harbour three tandem thioredoxin (Trx)-like domains, which are often found in proteins involved in ER quality control. Furthermore, we determined the three-dimensional structure of the third Trx-like domain, which exhibits an extensive hydrophobic patch concealed by its flexible C-terminal helix. Our structural data suggest that this hydrophobic patch is involved in intermolecular interactions, thereby contributing to the folding-sensor mechanism of UGGT.

In eukaryotic cells, proteins destined for the secretory pathway are translocated to the endoplasmic reticulum (ER) for folding, assembly and post-translational modification, including asparagine-linked glycosylation. To guarantee that only correctly folded glycoproteins are transported to the Golgi apparatus, the ER possesses a sophisticated protein quality control system^1^,^2^,^3^,^4^,^5^,^6^,^7^. In this system, *N*-linked oligosaccharides displayed on polypeptide chains function as quality tags for the determination of glycoprotein fates, i.e. folding, transport or degradation, that are selectively recognized by certain intracellular lectins^2^,^4^,^5^,^6^.

In the ER, newly synthesized proteins are cotranslationally modified with high mannose-type tetradecasaccharide (Glc_3_Man_9_GlcNAc_2_), which contains three non-reducing terminal branches (designated D1, D2 and D3)^8^. The D1 branch is capped with the triglucosyl moiety Glc-α1,2-Glc-α1,3-Glc. Glucosidase I removes the outermost α1,2-linked glucose from the D1 branch of this triantennary glycan^9^,^10^. Subsequently, glucosidase II trims the second and third α1,3-linked glucose residues^7^,^9^,^11^. The monoglucosylated D1 branch, an intermediate generated during this process, exhibits a critical determinant recognized by oxidoreductase (ERp57)-associated lectins, i.e. calnexin (CNX) and/or calreticulin (CRT). UDP-glucose:glycoprotein glucosyltransferase (UGGT) catalyzes reglucosylation, thereby regenerating monoglucosylated glycoforms, which are able to revisit the chaperone complex^7^,^12^,^13^,^14^,^15^,^16^,^17^,^18^. This glucose-trimming and -tagging process is called the ‘CNX/CRT cycle’.

UGGT acts as the gatekeeper in this system because this enzyme is capable of sensing the folding states of glycoproteins as potential substrates. UGGT only transfers monoglucose residues to incompletely folded glycoproteins^7^,^12^,^13^,^14^. UGGT is a large enzyme, comprising approximately 1500 amino acid residues, which has been putatively divided into two regions: an N-terminal folding-sensor region, which accounts for approximately 80% of the enzyme and is not homologous with any known structures, and a C-terminal catalytic domain, which accounts for the remaining 20% of the enzyme and belongs to the glycosyltransferase 8 family^19^,^20^. However, no further structural information is available on this key enzyme to date. Thus, the structural basis of the working mechanism of the CNX/CRT cycle remains unclear.

In this study, to elucidate the working mechanism of UGGT, we attempted to characterize the three-dimensional (3D) structure of its N-terminal folding-sensor region. We selected *Chaetomium thermophilum,* a thermophilic fungus, which survives at temperatures of up to 60°C^21^, as the source organism for the structural study of UGGT. Our bioinformatics analyses predicted that the folding-sensor region of UGGT contains three tandem thioredoxin (Trx)-like domains. Moreover, we determined the 3D structure of a Trx domain of UGGT, thereby providing structural insights into the mechanism of substrate recognition of this folding-sensor enzyme.

## Results

### Bioinformatic identification of three tandem Trx-like domains in folding sensor region of UGGT

**Figure 1:**
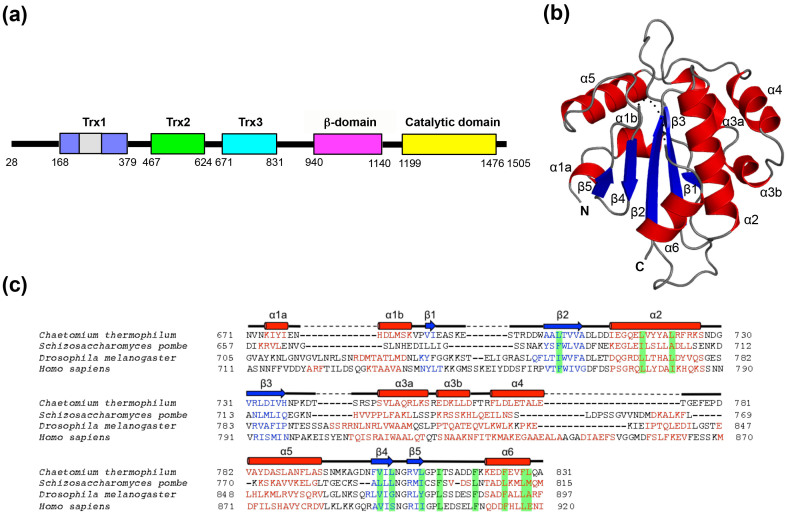
Crystal structure of the Trx3 domain of UGGT (a) Domain structure of *C. thermophilum* UGGT. The Trx3 domain (residues Asn671–Ala831) was crystallized in this study. (b) Ribbon models of the Trx3 domain of *C. thermophilum* UGGT (Form 1). The secondary structures are highlighted (α-helix, red; β-sheet, blue) and the linker regions are shown in grey. The positions of the N- and C-termini are also indicated. Dotted line indicates disorder segment. (c) Structure-based sequence alignment of the Trx3 domains of UGGT among species (from fungi to human). The secondary structures of the Trx3 domain of *C. thermophilum* UGGT are indicated above the amino acid sequence. The secondary structure elements (α-helix and β-sheet) were predicted using the program PROMALS3D^48^ and are highlighted in red and blue, respectively. Residues involving the C-terminal α6 helix or detergent interactions are highlighted in green.

To investigate the structure of the N-terminal folding-sensor region of UGGT, we subjected its amino acid sequence (residues 28–1198) to bioinformatics analysis using the programs PSIPRED^22^ and DISOPRED2^23^. The results indicate that the folding-sensor region of UGGT exhibits well-formed secondary structures: a mixed α/β region in the N-terminal part (residues 28–939) and a β-strand-rich region (termed the β-domain, residues 940–1140) around the C-terminus (Fig. 1a and Supplemental Fig. S1). Although the sequence homology of UGGT was modestly low (32.0%–34.5% identities) between the thermophilic fungus and humans (Supplemental Table S1), the secondary structure distributions appeared highly conserved across species. A remarkably disordered segment was identified at the connection between the β- and C-terminal catalytic domains (Supplemental Fig. S1). This structural feature is consistent with previously reported results of limited proteolysis^20^.

Next, we attempted to identify structural domain(s) within the N-terminal folding-sensor region using InterPro^24^ and Phyre2^25^. Regarding the β-domain, no significantly homologous domains were identified. On the other hand, the folding-sensor region of UGGT was found to harbour three tandem Trx-like domains: Trx1 (residues 168–379), Trx2 (residues 467–624) and Trx3 (residues 671–831) (Fig. 1 and Supplemental Fig. S1). The arrangement of these domains is essentially identical across species, suggesting that the common structural architecture of UGGT is evolutionarily conserved. Nonetheless, the three tandem Trx-like domains share relatively low sequence identities (Trx1 *versus* Trx2, 22.1%; Trx1 *versus* Trx3, 23.3%; Trx2 *versus* Trx3, 16.2% in *C. thermophilum*), suggesting variability in their three-dimensional structures.

### Crystal structure of the third Trx-like domain of UGGT

Based on the bioinformatic prediction that folding-sensor region of UGGT possesses three tandem Trx-like domains, we performed bacterial expression, purification and crystallization of a series of Trx domains. First, we expressed each of the three Trx domains. Although we were able to express the Trx3 domain as a soluble protein, the Trx1 and Trx2 domains formed inclusion bodies in *Escherichia coli* cells. Therefore, we made tandem constructs for their expression. Consequently, we were able to express Trx1-Trx2, Trx2-Trx3 and Trx1-Trx2-Trx3 proteins in their soluble form. Of these constructs, we successfully crystallized the Trx3 domain with the optimization of its N- and C-terminal sequences (residues 671–831), based on the identification of proteolytically stable fragments. However, despite extensive trials, we were unable to obtain crystals of the tandem constructs Trx1-Trx2, Trx2-Trx3 or Trx1-Trx2-Trx3.

We determined two forms of the crystal structure of Trx3 domain at 3.4 and 1.7 Å resolutions. The final model of Form 1, refined to a resolution of 3.40 Å, had an *R*_work_ of 23.5% and *R*_free_ of 29.2% (Table 1). The crystal belonged to space group *I*23 with six molecules per asymmetric unit. The structures of molecules A–F were highly similar to each other with an RMSD value of 0.11–0.37 Å for superimposed Cα atoms 94–155. Molecule A in the crystal structure, which had the lowest average *B* value (Table 1), was used for the comparative analysis and will be primarily described hereafter. On the other hand, Form 2 of the Trx3 domain of UGGT cocrystallized with a detergent ANAPOE C12E8 belonged to space group *C*222_1_ and diffracted up to 1.70-Å resolution. In the crystal structure, one molecule was contained per asymmetric unit. The final model of Form 2 had an *R*_work_ of 20.1% and *R*_free_ of 24.6% (Table 1).

As expected from the bioinformatics analysis, the crystal structure displayed a typical Trx-like fold, i.e. a five-stranded β-sheet with a β1–β3–β2–β4–β5 arrangement surrounded by six α-helices (Fig. 1b and 1c). In the crystal structure, a part of β5–α6 loop (residues 816–818) was disordered. The C-terminal α6-containing segment showed a higher crystallographic *B*-factor (87.7 Å^2^) than the average value (79.7 Å^2^; Table 1). Comparison of the structure of the Trx3 domain of UGGT with known protein structures using the DALI server revealed that the protein disulfide bond isomerase (DsbA/C) homologue, *Salmonella enterica* ScsC^26^, was the most structurally similar protein (*Z*-score = 9.4; RMSD = 2.9 Å; identify = 18.5%; PDB code: 4GXZ). As representative of the DsbA/C structure, the well-characterized crystal structure of *E. coli* DsbC (PDB code: 1EEJ)^27^ is also shown in Supplemental Figure 2. The overall fold of Trx3 domain of UGGT was essentially identical to that of ScsC except for their variable α helical segments between 3 and 4 (α3 and α4 in UGGT-Trx3 and α3–α5 in ScsC) (Supplemental Fig. S2b). DsbC also share very similar fold with the UGGT Trx3 domain except for the N-terminal α1 helix, which directly follows the dimerization domain in DsbC, and variable α3/α4 helices (Supplemental Fig. S2c). Compared with the crystal structure of the *E. coli* thioredoxin trxA^28^ (PDB code: 2TRX; Supplemental Fig. S2d), which exhibits typical Trx fold, three contiguous helical insertions, α3, α4 and α5, were identified between β3 and β4, as observed in DsbC^27^. Furthermore, an N-terminal segment containing α1 and β1 regions of the Trx3 domain of UGGT was significantly different from that of *E. coli* trxA^28^ in terms of topological arrangement. In the folds shared by the Trx3 domain of UGGT, ScsC and DsbC, α1 precedes β1, which makes anti-parallel β-strands with β3 (Supplemental Fig. S2a–c). In contrast, α1 was inserted between β1 and β2, both of which were parallel with respect to β3 (Supplemental Fig. S2d). In addition, our homology modeling suggest that the Trx1 and Trx2 domains exhibit typical Trx-like folds similar to the Trx3 domain and its structural homologs, except for the N-terminal and variable α helical segments between 3 and 4 and an insertion loop (residues 226–293) in Trx1 (Supplemental Fig. S3).

**Figure 2:**
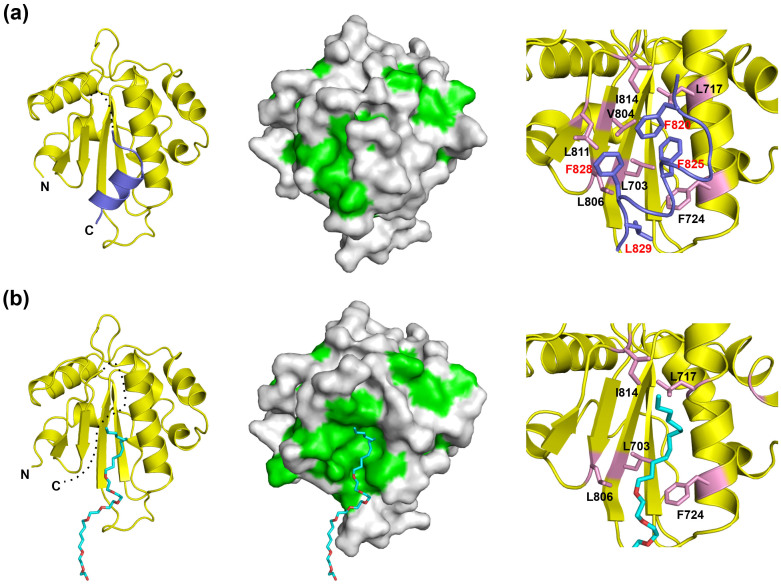
An extensive hydrophobic patch of the Trx3 domain is concealed by a flexible C-terminal helix. The crystal structures of the Trx3 domain in Forms 1 and 2 are indicated in (a) and (b), respectively. The ribbon and surface models are shown in the left and centre. Dotted lines indicate disordered segments. In the surface model (centre), the hydrophobic residues are shown in green. Close-up views of the C-terminal helix or detergent-interacting regions are represented on the right. Residues involved in these interactions are highlighted in the pink stick model. In Form 1 (a), the C-terminal α6 helix is highlighted in slate. In Form 2 (b), the detergent ANAPOE C12E8 is shown as a stick model.

The C-terminal α6 helix, which is followed by a putatively flexible linker region in UGGT, was completely disordered in the crystal structure of Form 2, suggesting the instability of this helix (Fig. 2b, left). Because of the absence of the α6 helix, an extensive hydrophobic patch was exposed on the surface of the Trx3 domain (Fig. 2b, centre). The detergent ANAPOE C12E8 was accommodated on this exposed hydrophobic patch. The α6 helix was stabilized mainly through its hydrophobic surface, containing Phe820, Phe825, Phe828 and Leu829, which made contact with the hydrophobic patch, including Leu703 (β2), Leu717, Phe724 (α2), Val804, Leu806 (β4), Leu811 (β5) and Ile814 (β5–α6 loop) (Fig. 2a, right). Most of these hydrophobic residues were involved in the interaction with the detergent in Form 2. Thus, the C-terminal α6 helix and detergent molecule occupy the common hydrophobic surface of the Trx3 domain. These hydrophobic residues are highly conserved among species (Fig. 1 and Supplemental Fig. S1).

## Discussion

In this study, we proposed that the folding-sensor region of UGGT contains three tandem Trx-like domains and, solved the first 3D structure of a structural domain, i.e. the third Trx-like domain, of this functional region (Fig. 1 and Supplemental Fig. S1). Trx-like domains are common to members of the protein disulfide isomerase (PDI) family, which are responsible for assisting protein folding in the ER^29^. Most PDI family members are multidomain proteins containing both redox-active and -inactive Trx-like domains in different arrangement^29^,^30^. For example, PDI (PDIA1) as a representative member of PDI family possesses four tandem Trx-like domains (designated *a*, *b*, *b*′ and *a*′), of which *a* and *a*′ domains have a CXXC catalytic motif, whereas *b* and *b*′ domains do not^31^,^32^. None of the Trx-like domains of UGGT possess the CXXC catalytic motif, indicating that this enzyme is not directly involved in thiol/disulfide exchange reactions. In this context, the *cis*-Pro loop adjacent to the CXXC motif, a hallmark of redox-active Trx-fold proteins^29^ and involved in substrate recognition in DsbA^32^, is not present in the Trx3 domain of UGGT. Noncatalytic Trx-like domains are often involved in substrate recognition^33^,^34^,^35^, co-factor interaction^36^ and functional intradomain interactions^34^. UGGT forms a stable complex with Sep15, a 15-kDa selenocystein-containing oxidoreductase^37^ which possesses one redox-active Trx-like domain and enhances the glucosyltransferase activity of UGGT^38^. It is plausible that Sep15 serves as a structural extension of UGGT with a complementary function.

Growing evidence implies that UGGT exhibits glucosyltransferase activity only against incompletely folded glycoproteins, suggesting that the folding-sensor region has exposed the hydrophobic patch as a principal substrate-binding site^7^,^12^,^13^,^14^. The Trx3 domain possesses an extensive hydrophobic patch, which is covered by the flexible C-terminal helix and can participate in interactions with hydrophobic molecules (Fig. 2). The hydrophobic residues involved in these intramolecular and intermolecular interactions are conserved across species (Supplemental Fig. S1). Thus, our crystallographic study provides an atomic view of the potential substrate-binding site of UGGT. In addition, our homology modeling data suggested that Trx1 and Trx2 domains also exhibit larger hydrophobic patches located at the opposite site as compared with that of the Trx3 domain, suggesting the possibility of their involvement in substrate recognition (Supplemental Fig. S4). Concomitantly, this may be the cause of inclusion body formation of the isolated Trx1 and Trx2 domains. In general, molecular chaperones undergo conformational transitions coupled with the shielding and exposure of their hydrophobic patches as substrate-binding sites^35^,^39^. Although we cannot exclude the possibility that the hydrophobic patch of the Trx3 domain is covered by other domain(s) in intact UGGT, the flexible properties of the C-terminal helix of Trx3 may contribute to regulatory mechanisms underlying the folding-sensing function of this domain.

In summary, our bioinformatic analyses predicted that the folding-sensor region of UGGT harbours three tandem Trx-like domains. Moreover, we provided snapshots of the 3D structure of the third Trx-like domain, in which a putative substrate-binding hydrophobic patch is intramolecularly masked or involved in an intermolecular interaction, offering a key breakthrough toward understanding of the functional mechanisms of this ER folding-sensor enzyme.

## Methods

### Protein expression and purification

*C. thermophilum* var. *thermophilum* La Touche (DSM 1495) was obtained from DSMZ, Braunschweig, Germany. Total RNA was isolated using TRIzol® reagent (Life Technologies). The cDNA was synthesized using SuperScript® III Reverse Transcriptase (Life Technologies) with oligo d(T) primers according to the manufacturer's instructions. Full-length UGGT cDNA was cloned by PCR using a *C. thermophilum* genomic DNA database^21^. Recombinant UGGT proteins were expressed as glutathione *S*-transferase (GST)-fused proteins. The Trx1 (residues 168–379), Trx2 (residues 467–624), Trx3 (residues 671–831), Trx1-Trx2 (residues 168–624), Trx2-Trx3 (residues 467–831) and Trx1-Trx2-Trx3 (residues 168–831) domains were amplified by PCR and subcloned into the *Bam*HI and *Xba*I sites of a modified pCold-GST vector (Takara Bio Inc.)^40^, in which the factor Xa site was replaced with the tobacco etch virus (TEV) protease recognition site. Recombinant proteins were expressed in *E. coli* BL21 Star™ cells (Life Technologies) according to the manufacturer's protocols (Takara Bio Inc.). GST-fused proteins were purified using glutathione-Sepharose™ columns (GE Healthcare). Subsequently, the GST tag was removed by adding TEV protease to the resin for 12 h at 277 K, leaving two additional residues Gly-Ser at the N-terminus. The resultant proteins were further purified by size-exclusion chromatography (Superdex-200; GE Healthcare) using a buffer containing 20 mM Tris-HCl (pH 7.5), 150 mM NaCl and 0.1 mM EDTA. The selenomethione (SeMet)-labelled Trx3 domain was expressed in *E. coli* B834 (DE3) using M9 minimal medium with SeMet. Expression and purification were performed following the same protocol as that for the native protein. Purified proteins were dialyzed against a buffer containing 10 mM Tris-HCl (pH 7.5) and 100 mM NaCl. The integrity of the protein samples was validated by matrix-assisted laser desorption/ionization time-of-flight mass spectrometry (MALDI-TOF/MS) analysis using an AXIMA-CFR™ spectrometer (Shimadzu) and N-terminal Edman sequencing with a Procise 494HT protein sequenator (ABI/Life Technologies).

### Protein crystallization, X-ray data collection and structure determination

The crystals of the Trx3 domain of UGGT (Form 1, 10 mg/ml) were grown in a buffer containing 60% Tacsimate (pH 7.0) for 2 weeks at 289 K. The crystals of the Trx3 domain of UGGT (Form 2) were obtained by equilibrating a solution of 8 mg/ml protein with 1.2 mM ANAPOE C12E8 (polyoxyethylene[8]dodecyl ether **·** 3,6,9,12,15,18,21,24-octaoxahexatriacontan-1-ol) mixed with an equal volume of precipitant solution containing 23% PEG3350, 0.1 M Tris-HCl (pH 7.0) and 0.2 M ammonium acetate for 6 days at 289 K. The crystals were transferred into the reservoir solution and flash-cooled in liquid nitrogen. Data sets for Forms 1 and 2 were collected using synchrotron radiation at 13B1 of the National Synchrotron Radiation Research Center (Hsinchu, Taiwan) and AR-NW12A of the Photon Factory (Tsukuba, Japan), respectively. All diffraction data were processed using HKL2000^41^. Crystal parameters are summarized in Table 1.

The 1.70 Å-resolution crystal structure of the Trx3 domain of UGGT (Form 2) was solved using the SAD method. The initial phase was determined using the SHELX C/D/E program^42^. The initial model was automatically built using ARP/wARP^43^. Further manual model building into the electron density maps and refinement were performed using COOT^44^ and REFMAC5^45^, respectively. The 3.40 Å-resolution structure of the Trx3 domain of UGGT (Form 1) was solved by molecular replacement using the program Phaser^46^ with the crystal structure of Form 2 as a search model. The stereochemical quality of the final model was assessed by RAMPAGE^47^. The final refinement statistics are summarized in Table 1. Graphic figures were prepared using PyMOL (http://www.pymol.org/). Homology modeling of the Trx1 and Trx2 domains were performed using Phyre2^25^ with *Neisseria gonorrhoeae* DsbC-like protein (PDB code: 3GV1) and *Neisseria meningitidis* DsbA1 (PDB code: 3DVW) as templates, respectively.

## Author Contributions

T.S. and K.K. conceived and designed the experiments; T.Z. and T.S. performed the bioinformatics analyses and crystallographic experiments; all authors wrote and reviewed the manuscript.

## Additional Information

**Accession codes** The coordinates and structural factors of the crystal structures of the Trx3 domain of *C. thermophilum* UGGT (Forms 1 and 2) have been deposited in the Protein Data Bank under the accession numbers 3WZT and 3WZS, respectively.

## Supplementary Material

Supplementary Information Supplementary information

## Figures and Tables

**Table 1:** Data collection and refinement statistics for UGGT-Trx3 domain

	Form 1	Form 2
**Crystallographic data**		
Space group	*I*23	*C*222_1_
Unit cell *a*/*b*/*c* (Å)	196.4/196.4/196.4	46.2/93.6/81.9
α/β/γ (°)	90.0/90.0/90.0	90.0/90.0/90.0
**Data processing statistics**		
Beam line	NSRRC 13B1	PF-AR NW12A
Wavelength (Å)	0.97888	0.97921
Resolution (Å)	50–3.40 (3.52–3.40)	50–1.70 (1.73–1.70)
Total/unique reflections	778,614/17,411	134,741/20,126
Completeness (%)	100.0 (100.0)	98.5 (98.9)
*R*_merge_ (%)	12.7 (67.7)	8.2 (36.6)
*I*/σ (*I*)	34.1 (6.7)	47.9 (7.2)
**Refinement statistics**		
Resolution (Å)	20.0–3.40	20.0–1.70
*R*_work_/*R*_free_ (%)	23.5/29.2	20.1/24.6
R.m.s. deviations from ideal		
Bond lengths (Å)	0.010	0.011
Bond angles (°)	1.28	1.47
Ramachandran plot (%)		
Favored	96.5	98.3
Allowed	3.5	1.7
Number of atoms		
Protein atoms (A/B/C/D/E/F)	1239/1246/1127/1231/738/871	1166
Water molecules	-	120
Detergent molecule	-	37
Average *B*-values (Å^2^)		
Protein atoms (A/B/C/D/E/F)	79.7/80.6/92.6/95.2/135.1/139.8	23.8
Water molecules	-	30.1
Detergent molecule	-	64.9
