# Supplementary material for: Structural insight into substrate recognition by the endoplasmic reticulum folding-sensor enzyme: crystal structure of third thioredoxin-like domain of UDP-glucose:glycoprotein glucosyltransferase
Source: Sci Rep. 2014 Dec 4;4:7322. doi: 10.1038/srep07322 (PMC4255179; doi:10.1038/srep07322)
Supplement: Supplementary Information [file srep07322-s1.pdf]

Supplementary information

Structural insight into substrate recognition by the endoplasmic reticulum folding-sensor enzyme: crystal structure of third thioredoxin-like domain of UDP-glucose:glycoprotein glucosyltransferase

Tong Zhu[1,2,3], Tadashi Satoh[3,4] and Koichi Kato[1,2,3]

[1]School of Physical Sciences, The Graduate University for Advanced Studies, 5-1 Higashiyama, Myodaiji, Okazaki, Aichi 444-8787, Japan; [2]Okazaki Institute for Integrative Bioscience and Institute for Molecular Science, National Institutes of Natural Sciences, 5-1 Higashiyama, Myodaiji, Okazaki, Aichi 444-8787, Japan; [3]Graduate School of Pharmaceutical Sciences, Nagoya City University, 3-1 Tanabe-dori, Mizuho-ku, Nagoya 467-8603, Japan; [4]JST, PRESTO, 3-1 Tanabe-dori, Mizuho-ku, Nagoya 467-8603, Japan;

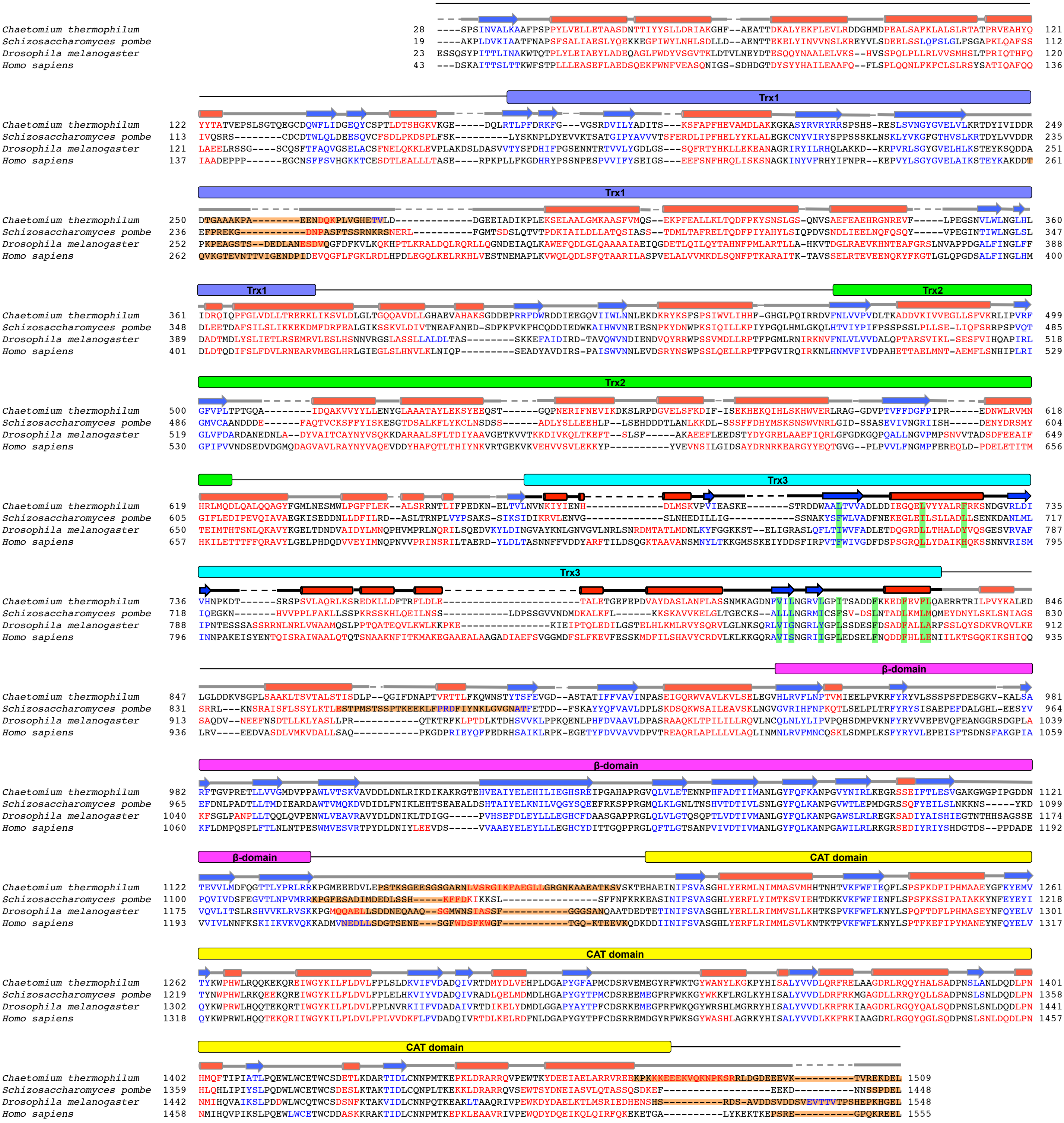

Fig. S1. Structure-based sequence alignment of full-length UGGT among species. The amino acid sequences of UGGT from fungi to human are aligned using the program PROMALS3D[49]. The predicted secondary structure elements are highlighted as in Fig. 1. Predicted disordered segments and residues involving the C-terminal α6 helix or detergent interactions are highlighted in orange and green, respectively.

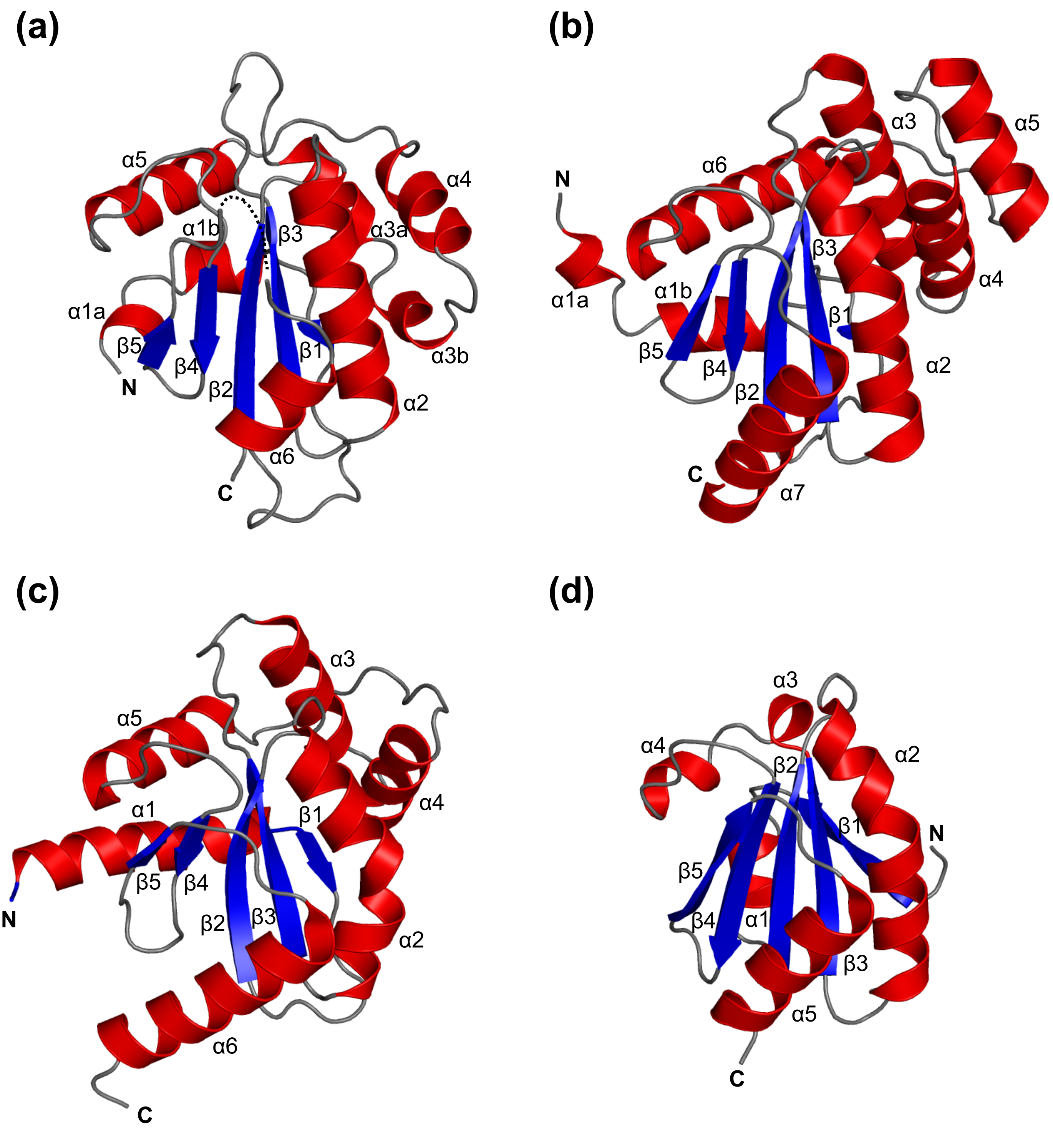

Fig. S2. Comparison of the Trx3 domain with homologous structures. (a) Trx3 domain of *C. thermophilum* UGGT (Form 1). (b) DsbA/C homologue, *Salmonella enterica* ScsC (PDB code: 4GXZ). (c) *E. coli* DsbC (PDB code: 1EEJ). For clarity, the N-terminal dimerization domain (residues 1–60) is not shown in the model. (d) *E. coli* thioredoxin (PDB code: 2TRX). The secondary structures are colored as in Fig. 1.

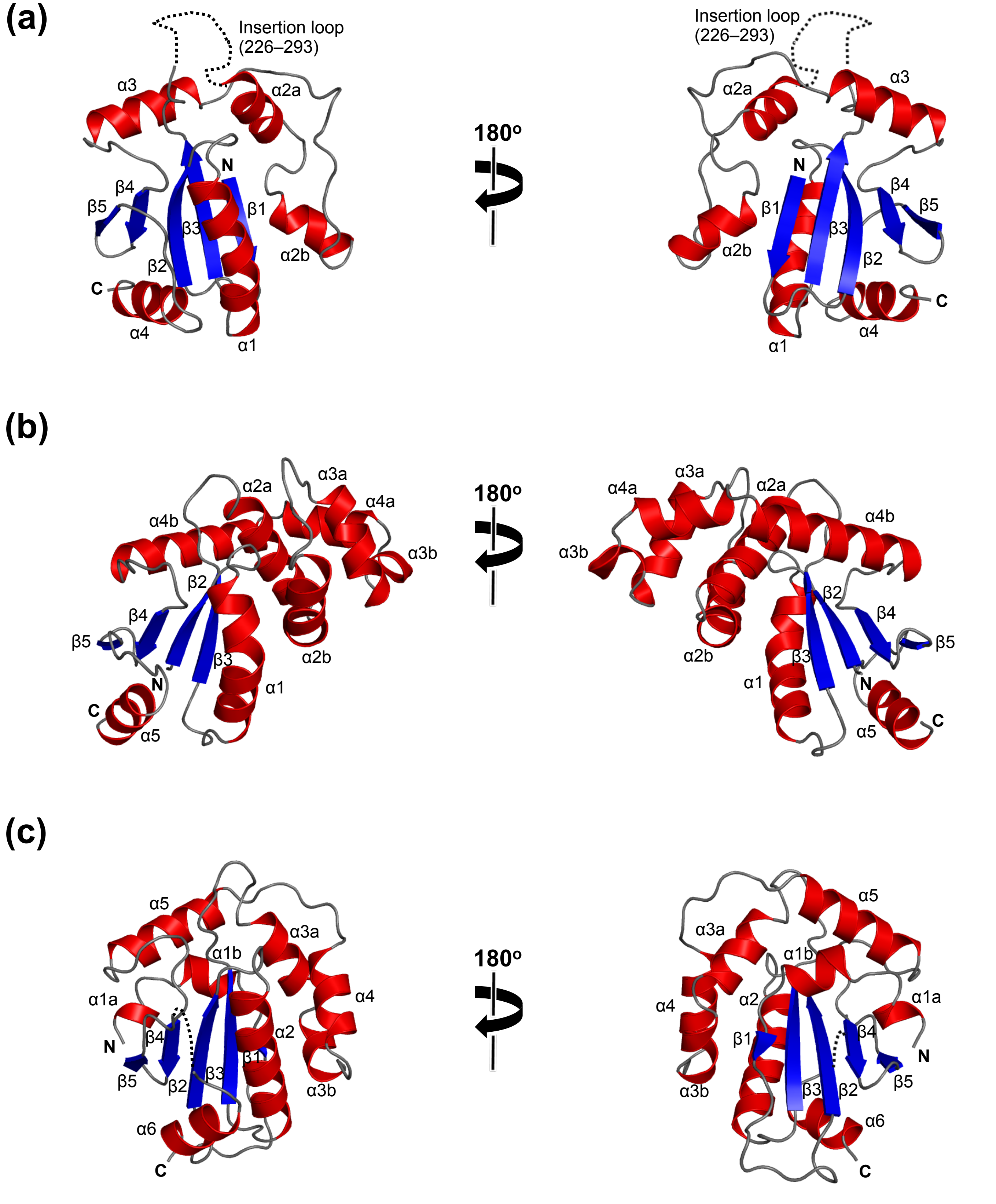

Fig. S3. Comparison of the overall structures of Trx-like domains in UGGT. (a) Homology model of the Trx1 domain (residues 168–379). An insertion loop (residues 226–293) is shown in dashed line. (b) Homology model of the Trx2 domain (residues 467–624). (c) Crystal structure of the Trx3 domain (residues 671–831, Form 1). The secondary structures are colored as in Fig. 1.

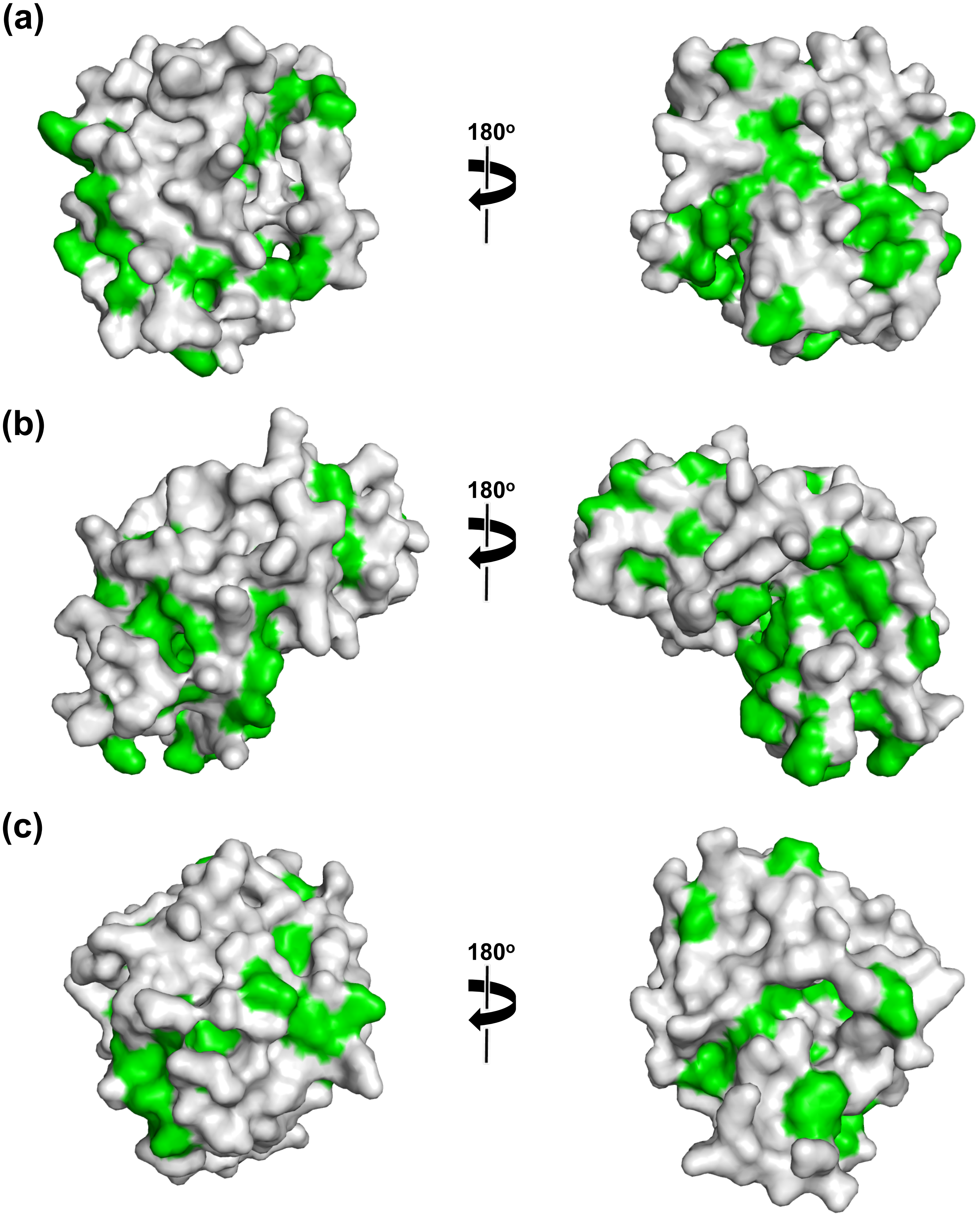

Fig. S4. Comparison of the Trx-like domains of UGGT in terms of surface hydrophobicity. (a) Homology model of the Trx1 domain. (b) Homology model of the Trx2 domain. (c) Crystal structure of the Trx3 domain (Form 1). The hydrophobic residues are colored green.

Table S1. Sequence identity of UGGT among species

	Chaetomium thermophilum	*Schizosaccharomyces pombe*	*Drosophila melanogaster*	*Homo sapiens*
	**Full-length (28−1509)**			
Chaetomium thermophilum	-	35.3%	32.0%	34.5%
Schizosaccharomyces pombe	35.3%	-	31.0%	29.9%
Drosophila melanogaster	32.0%	31.0%	-	44.2%
Homo sapiens	34.5%	29.9%	44.2%	-
	**Folding sensor region (28−1198)**			
Chaetomium thermophilum	-	26.8%	24.6%	26.7%
Schizosaccharomyces pombe	26.8%	-	22.6%	20.8%
Drosophila melanogaster	24.6%	22.6%	-	37.0%
Homo sapiens	26.7%	20.8%	37.0%	-
	**Catalytic domain (1199−1509)**			
Chaetomium thermophilum	-	67.3%	58.5%	64.0%
Schizosaccharomyces pombe	67.3%	-	60.7%	63.7%
Drosophila melanogaster	58.5%	60.7%	-	73.9%
Homo sapiens	64.0%	63.7%	73.9%	-
